# Modelling the impact of intermittent preventive treatment for malaria on selection pressure for drug resistance

**DOI:** 10.1186/1475-2875-6-9

**Published:** 2007-01-22

**Authors:** Neal Alexander, Colin Sutherland, Cally Roper, Badara Cissé, David Schellenberg

**Affiliations:** 1 Department of Infectious and Tropical Diseases, London School of Hygiene and Tropical Medicine, Keppel Street, London WC1E 7HT, UK; 2 Institut de Recherche pour le Développement, UR 077, Dakar, Sénégal

## Abstract

**Background:**

Intermittent preventive treatment (IPT) is a promising intervention for malaria control, although there are concerns about its impact on drug resistance.

**Methods:**

The key model inputs are age-specific values for a) baseline anti-malarial dosing rate, b) parasite prevalence, and c) proportion of those treated with anti-malarials (outside IPT) who are infected. These are used to estimate the immediate effect of IPT on the genetic coefficient of selection (*s*). The scenarios modelled were year round IPT to infants in rural southern Tanzania, and three doses at monthly intervals of seasonal IPT in Senegal.

**Results:**

In the simulated Tanzanian setting, the model suggests a high selection pressure for drug resistance, but that IPTi would only increase this by a small amount (4.4%). The percent change in *s *is larger if parasites are more concentrated in infants, or if baseline drug dosing is less common or less specific. If children aged up to five years are included in the Tanzanian scenario then the predicted increase in *s *rises to 31%. The Senegalese seasonal IPT scenario, in children up to five years, results in a predicted increase in *s *of 16%.

**Conclusion:**

There is a risk that the useful life of drugs will be shortened if IPT is implemented over a wide childhood age range. On the other hand, IPT delivered only to infants is unlikely to appreciably shorten the useful life of the drug used.

## Background

Intermittent preventive treatment (IPT) for malaria is the administration of a therapeutic course of an anti-malarial to people who are at risk, regardless of their current infection status[1].

Trials in African locations of intermittent preventive treatment in infants (IPTi) against clinical malaria have yielded estimates of efficacy in the range 20–60% [2-5]. However, there are concerns that IPT could foster drug resistance [6-8], repeating the history of previous control methods which administered anti-malarials to people who were not malaria cases[1]. Selection pressure for resistance depends not only on the drug dosing rate, but also on the infection status of the recipients[9].

Some drug resistance models may meet their objectives without explicitly including age structure[10,11]. However, the use of IPT in children is, by definition, dependent on age. Moreover, both drug dosing rate and infection status are likely to vary significantly in children according to their age and immune status. These considerations led us to devise a simple model for the genetic coefficient of selection (*s*) for a drug resistance associated gene, or linked set of genes.

## Methods

### Model definition

The spread of a drug-resistant genotype can be expressed in terms of the selection coefficient *s*, which is the relative difference in reproductive success. Following Brown and Rothery [12], but for *Plasmodium *rather than a diploid organism, the (relative) frequency of the resistant genotype is expected to increase logistically with parameter *s*[13]. Logistic growth is approximately exponential growth at small relative frequencies, then approximately linear at relative frequencies around 50%, and finally asymptotically approaches 100%. The selection coefficient *s *is modelled as a function of: age-dependent parasite prevalence *p*(*a*); prevalence *π*(*a*) of a history of taking anti-malarials in the previous *d *days; and positive predictive value *ψ*(*a*) of anti-malarial treatment, i.e. the proportion of those treated with anti-malarials (outside of IPT) who were, in fact, infected. In the general population, the dosing rate is *λ*(*a*) = -log(1-*π*(*a*))/*d*, and in parasite positive people it is *λ*_+_(*a*) = -*ψ*(*a*) log(1-*π*(*a*))/(*p*(*a*)*d*).

The following form was chosen to represent parasite prevalence:

p(a)=AA−B(e−Ba−e−Aa+C(1−e−Aa))     1)
 MathType@MTEF@5@5@+=feaafiart1ev1aaatCvAUfKttLearuWrP9MDH5MBPbIqV92AaeXatLxBI9gBaebbnrfifHhDYfgasaacH8akY=wiFfYdH8Gipec8Eeeu0xXdbba9frFj0=OqFfea0dXdd9vqai=hGuQ8kuc9pgc9s8qqaq=dirpe0xb9q8qiLsFr0=vr0=vr0dc8meaabaqaciaacaGaaeqabaqabeGadaaakeaacqWGWbaCcqGGOaakcqWGHbqycqGGPaqkcqGH9aqpdaWcaaqaaiabdgeabbqaaiabdgeabjabgkHiTiabdkeacbaadaqadaqaaiabdwgaLnaaCaaaleqabaGaeyOeI0IaemOqaiKaemyyaegaaOGaeyOeI0Iaemyzau2aaWbaaSqabeaacqGHsislcqWGbbqqcqWGHbqyaaGccqGHRaWkcqWGdbWqdaqadaqaaiabigdaXiabgkHiTiabdwgaLnaaCaaaleqabaGaeyOeI0IaemyqaeKaemyyaegaaaGccaGLOaGaayzkaaaacaGLOaGaayzkaaGaaCzcaiaaxMaacqaIXaqmcqGGPaqkaaa@4F7C@

This function *p*(*a*) increases from zero at age *a *= 0 to reach its maximum value at age *a *= log(*B*/(*A*(1+*C*)))/(*B*-*A*), with an asymptote at large *a *of *CA*/(*A*-*B*). For the drug history *π*(*a*), and positive predictive value *ψ*(*a*), logistic functions are used, with the form:

U+(L−U)eα+βa1+eα+βa     2)
 MathType@MTEF@5@5@+=feaafiart1ev1aaatCvAUfKttLearuWrP9MDH5MBPbIqV92AaeXatLxBI9gBaebbnrfifHhDYfgasaacH8akY=wiFfYdH8Gipec8Eeeu0xXdbba9frFj0=OqFfea0dXdd9vqai=hGuQ8kuc9pgc9s8qqaq=dirpe0xb9q8qiLsFr0=vr0=vr0dc8meaabaqaciaacaGaaeqabaqabeGadaaakeaacqWGvbqvcqGHRaWkcqGGOaakcqWGmbatcqGHsislcqWGvbqvcqGGPaqkdaWcaaqaaiabdwgaLnaaCaaaleqabaacciGae8xSdeMaey4kaSIae8NSdiMaemyyaegaaaGcbaGaeGymaeJaey4kaSIaemyzau2aaWbaaSqabeaacqWFXoqycqGHRaWkcqWFYoGycqWGHbqyaaaaaOGaaCzcaiaaxMaacqaIYaGmcqGGPaqkaaa@468C@

where *U *and *L *are the upper and lower asymptotes respectively, *ã *= -*α*/*β *is the age at which the midpoint of *U *and *L *is attained, and *β *measures the steepness of the transition. Note that gene frequency is expected to follow a logistic function of time on the basis of population genetic theory[12], whereas logistic functions of age are used for drug history (*π*) and positive predictive value (*ψ*) because they approximate empirically the patterns seen in the data sources used (see below, subsection 'Parameter values'). The implementation of IPT, at a given rate of doses per year, is included by adding this rate to the pre-IPT dosing rates. This completes the specification of the dosing rates *λ*(*a*) and *λ*_+_(*a*).

The fitness of an inoculated parasite – or the number of next-generation infections to which it gives rise – is, by default, assumed to be proportional to the duration *D *of infection in the host. However, in sensitivity analysis, the infectivity was allowed to decrease, exponentially, at various rates, over the duration of the infection. In the absence of drug, a default value of 3 months is used for *D*[9,14]. In the presence of drug, it is necessary to work out the expected duration *D' *<*D *of a sensitive infection. This is done as the probability-weighted sum over three cases. First, the drug may be present at an effective level at the time of parasite inoculation, with probability 1 - exp(-*λ*(*a*)*d'*), where *d' *is the number of days for which the drug retains its killing effect (default value 14 days, representing sulfadoxine-pyrimethamine or SP). In this case the duration of the infection is zero. Second, the infection may persist, unaffected by drug ingestion, for its natural duration *D*, with probability 1 - exp(-*λ*(*a*)*d'*) exp(-*λ*_+_(*a*)*D*). Third, the drug is not present on inoculation, but is taken before the infection ends naturally. The expected value of infection duration is then the integral ∫0Dtλ+(a)e−λ+(a)tdt=(1/λ+(a))−e−λ+(a)D(D+(1/λ+(a)))
 MathType@MTEF@5@5@+=feaafiart1ev1aaatCvAUfKttLearuWrP9MDH5MBPbIqV92AaeXatLxBI9gBaebbnrfifHhDYfgasaacH8akY=wiFfYdH8Gipec8Eeeu0xXdbba9frFj0=OqFfea0dXdd9vqai=hGuQ8kuc9pgc9s8qqaq=dirpe0xb9q8qiLsFr0=vr0=vr0dc8meaabaqaciaacaGaaeqabaqabeGadaaakeaadaWdXaqaaiabdsha0HGaciab=T7aSnaaBaaaleaacqGHRaWkaeqaaaqaaiabicdaWaqaaiabdseaebqdcqGHRiI8aOGaeiikaGIaemyyaeMaeiykaKIaemyzau2aaWbaaSqabeaacqGHsislcqWF7oaBdaWgaaadbaGaey4kaScabeaaliabcIcaOiabdggaHjabcMcaPiabdsha0baakiabbsgaKjabdsha0jabg2da9maabmaabaGaeGymaeJaei4la8Iae83UdW2aaSbaaSqaaiabgUcaRaqabaGccqGGOaakcqWGHbqycqGGPaqkaiaawIcacaGLPaaacqGHsislcqWGLbqzdaahaaWcbeqaaiabgkHiTiab=T7aSnaaBaaameaacqGHRaWkaeqaaSGaeiikaGIaemyyaeMaeiykaKIaemiraqeaaOWaaeWaaeaacqWGebarcqGHRaWkdaqadaqaaiabigdaXiabc+caViab=T7aSnaaBaaaleaacqGHRaWkaeqaaOGaeiikaGIaemyyaeMaeiykaKcacaGLOaGaayzkaaaacaGLOaGaayzkaaaaaa@657B@. Adding the three expected values yields the expected duration *D' *as a function of age:

D′(a)=e−λ(a)d′(De−λ+(a)D+(1−e−λ+(a)D)(1λ+(a)−e−λ+(a)D(D+1λ+(a))))     3)
 MathType@MTEF@5@5@+=feaafiart1ev1aaatCvAUfKttLearuWrP9MDH5MBPbIqV92AaeXatLxBI9gBaebbnrfifHhDYfgasaacH8akY=wiFfYdH8Gipec8Eeeu0xXdbba9frFj0=OqFfea0dXdd9vqai=hGuQ8kuc9pgc9s8qqaq=dirpe0xb9q8qiLsFr0=vr0=vr0dc8meaabaqaciaacaGaaeqabaqabeGadaaakeaacuWGebargaqbaiabcIcaOiabdggaHjabcMcaPiabg2da9iabdwgaLnaaCaaaleqabaGaeyOeI0ccciGae83UdWMaeiikaGIaemyyaeMaeiykaKIafmizaqMbauaaaaGcdaqadaqaaiabdseaejabdwgaLnaaCaaaleqabaGaeyOeI0Iae83UdW2aaSbaaWqaaiabgUcaRaqabaWccqGGOaakcqWGHbqycqGGPaqkcqWGebaraaGccqGHRaWkdaqadaqaaiabigdaXiabgkHiTiabdwgaLnaaCaaaleqabaGaeyOeI0Iae83UdW2aaSbaaWqaaiabgUcaRaqabaWccqGGOaakcqWGHbqycqGGPaqkcqWGebaraaaakiaawIcacaGLPaaadaqadaqaamaalaaabaGaeGymaedabaGae83UdW2aaSbaaSqaaiabgUcaRaqabaGccqGGOaakcqWGHbqycqGGPaqkaaGaeyOeI0Iaemyzau2aaWbaaSqabeaacqGHsislcqWF7oaBdaWgaaadbaGaey4kaScabeaaliabcIcaOiabdggaHjabcMcaPiabdseaebaakmaabmaabaGaemiraqKaey4kaSYaaSaaaeaacqaIXaqmaeaacqWF7oaBdaWgaaWcbaGaey4kaScabeaakiabcIcaOiabdggaHjabcMcaPaaaaiaawIcacaGLPaaaaiaawIcacaGLPaaaaiaawIcacaGLPaaacaWLjaGaaCzcaiabiodaZiabcMcaPaaa@73B0@

The total fitness of a parasite genotype is proportional to the number of next-generation parasites, integrated over the whole human age range. The contribution of small age interval, *a *to *a*+*δa*, is proportional to: i) *D'*(*a*) (equation3); ii) the number of people in the interval; iii) the proportion of them who are parasitaemic, ie *p*(*a*) (equation1); and iv) the width of the interval *δa*. Assuming Type II mortality, ie a population pyramid which reduces exponentially with age, the number of people in the age interval is proportional to exp(-*a*/a¯
 MathType@MTEF@5@5@+=feaafiart1ev1aaatCvAUfKttLearuWrP9MDH5MBPbIqV92AaeXatLxBI9gBaebbnrfifHhDYfgasaacH8akY=wiFfYdH8Gipec8Eeeu0xXdbba9frFj0=OqFfea0dXdd9vqai=hGuQ8kuc9pgc9s8qqaq=dirpe0xb9q8qiLsFr0=vr0=vr0dc8meaabaqaciaacaGaaeqabaqabeGadaaakeaacuWGHbqygaqeaaaa@2E0F@), where a¯
 MathType@MTEF@5@5@+=feaafiart1ev1aaatCvAUfKttLearuWrP9MDH5MBPbIqV92AaeXatLxBI9gBaebbnrfifHhDYfgasaacH8akY=wiFfYdH8Gipec8Eeeu0xXdbba9frFj0=OqFfea0dXdd9vqai=hGuQ8kuc9pgc9s8qqaq=dirpe0xb9q8qiLsFr0=vr0=vr0dc8meaabaqaciaacaGaaeqabaqabeGadaaakeaacuWGHbqygaqeaaaa@2E0F@ is the mean age in years (default 15 years). It was assumed that no malaria infections, and no IPT dosing, occur below a minimum age *a*_0_, default value 2 months. Therefore the fitness is proportional to ∫a0∞D′(a)p(a)e−a/a¯da
 MathType@MTEF@5@5@+=feaafiart1ev1aaatCvAUfKttLearuWrP9MDH5MBPbIqV92AaeXatLxBI9gBaebbnrfifHhDYfgasaacH8akY=wiFfYdH8Gipec8Eeeu0xXdbba9frFj0=OqFfea0dXdd9vqai=hGuQ8kuc9pgc9s8qqaq=dirpe0xb9q8qiLsFr0=vr0=vr0dc8meaabaqaciaacaGaaeqabaqabeGadaaakeaadaWdXaqaaiqbdseaezaafaGaeiikaGIaemyyaeMaeiykaKIaemiCaaNaeiikaGIaemyyaeMaeiykaKIaemyzau2aaWbaaSqabeaacqGHsislcqWGHbqycqGGVaWlcuWGHbqygaqeaaaaaeaacqWGHbqydaWgaaadbaGaeGimaadabeaaaSqaaiabg6HiLcqdcqGHRiI8aOGaeeizaqMaemyyaegaaa@43D1@. This integral is evaluated numerically. The reproductive success of resistant parasites is calculated by setting the dosing rate to zero, and *s *is obtained as one minus the ratio of fitness of sensitive to resistant parasites.

In areas of seasonal transmission, a higher maximum age of IPT may be used, with dosing concentrated in the transmission season. Here, *s *was estimated via a weighted sum of the fitness in each season.

In this simple model parasite prevalence appears only as an input, not an output. So IPT does not feed back into prevalence, and the effects on selection pressure are those immediately following the introduction of IPT.

### Parameter values

**Figure 1:**
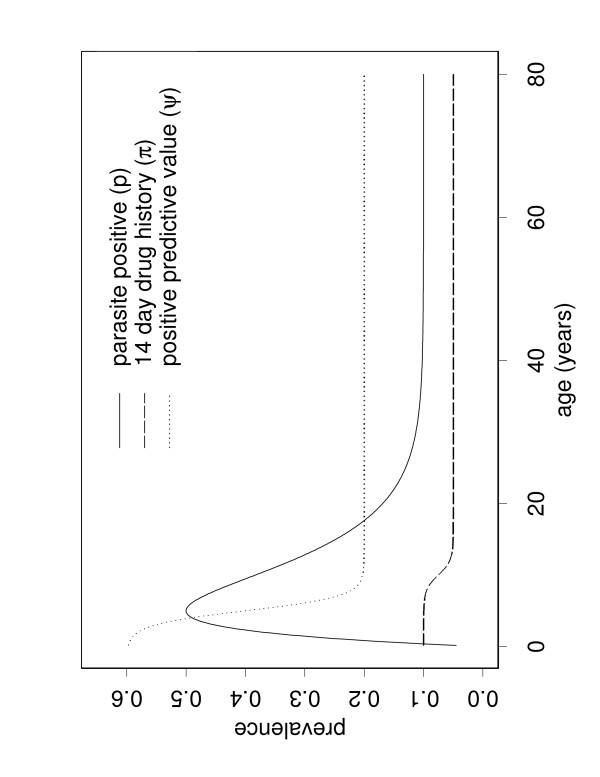
**Model inputs. **Default prevalence curves, illustrating the default parameter values for the proportion of people slide positive (*p*, solid line), the proportion with a 14 day history of taking anti-malarials (*π*, dashed line), and the positive predictive value of (*ψ*, dotted line).

The key input parameters were chosen for rural southern Tanzania. Unpublished data from surveys by Armstrong Schellenberg and colleagues[15] estimated that the 14-day history of anti-malarial dosing *π *(*a*) decreased from approximately 10% in young children to 5% in adults. Parasite prevalence *p*(*a*) was chosen to approximately reflect the situation in the same location, and was assumed to peak at 50% at age 5 years, decreasing to 10% in adults. The positive predictive value of treatment *ψ *(*a*) was assumed to decrease from 60% in children to 20% in adults[16]. The following values were used: *β *= -1, and *ã *= 10 years for *π*(*a*), 5 years for *ψ*(*a*). Default values for *p*(*a*), *π*(*a*) and *ψ*(*a*) are shown in Figure 1.

Parameters for the seasonal IPT scenario, in children up to five years, were chosen to reflect Niakhar, Senegal. Peak parasite prevalence was set at age 7 years, and was 90% in the three month high transmission, otherwise 40%. The asymptotes of *π*(*a*) at low and high ages were 33% and 12% respectively in the high transmission season; otherwise 5% and 2%. The corresponding values for *ψ*(*a*) were 95% and 85% in the transmission season, otherwise 50% and 20%. Other parameters were unchanged.

## Results

In the simulated Tanzanian setting, the selection coefficient for resistant parasites in the absence of IPT is estimated at 0.267, and in the presence of IPTi is 0.279, an increase of 4.4%. So resistant parasites are expected to yield, on average, about 27% more offspring than sensitive ones, but IPTi makes a negligible impact on this.

**Figure 2:**
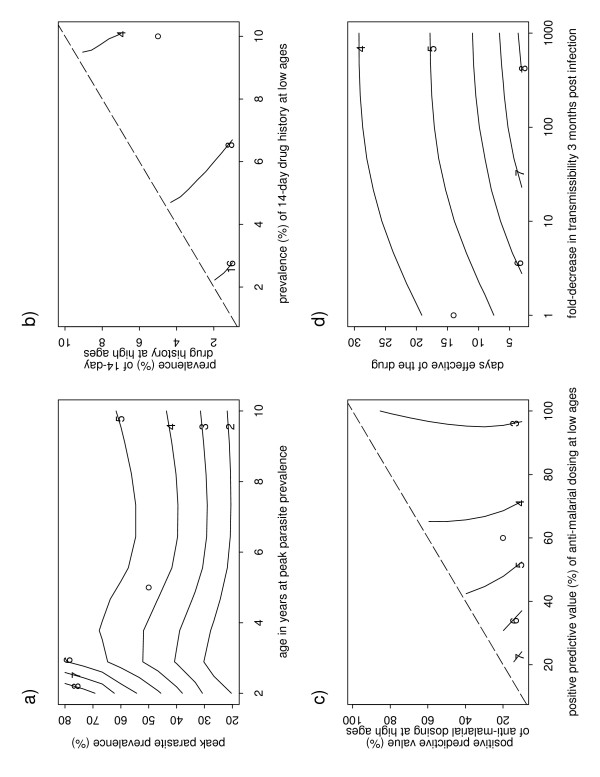
**Sensitivity analysis of main parameters**. Each panel shows contours of the percent change in selection coefficient *s*, as a function of two parameters. The default parameter values (Tanzanian setting), and predicted change in *s*, are shown by ○ in each panel. Parts of panels b) and c) are blank because of the assumption that the drug history, and positive predictive value of treatment, would be higher in young children than adults.

Sensitivity analysis of the default parameters is shown in Figure 2, with each panel showing contours of the estimated percent change in the selection coefficient (*s*) when a selected pair of parameters is varied, with the ○ symbol shows the default values. Since the default values give a change in *s *of 4.4%, the ○ symbol lies between the contours of 4% and 5% (or between 4 and 8%, in panel b)). Panel a) shows the strongest predicted effect on *s *occurs when peak parasitaemia prevalence is high, and occurs in younger children (i.e. towards the left and top of the graph). Panel b) show the percent change in *s *would, as expected, be greater if the background dosing rate were less. Panel c) shows that the effect on *s *is greater if the positive predictive value of the non-IPT dosings is less. This can be understood as follows. If the non-IPT dosings are being directed less efficiently to those infected, then the impact on parasites is less, but, when IPT is introduced, the percent change in *s *is greater. Paneld) shows that the percent change in *s *is slightly lower for drugs which are effective for longer, probably because of greater overlap of IPT and non-IPT dosings. Panel d) also shows that the effect on *s *of the rate of decrease of transmissibility is very small. The percent change in *s *is also slightly larger if the natural duration of infection is shorter. Within the plausible ranges examined, the background dosing rate is the most important determinant of the predicted impact of IPT on selection pressure, leading to increases in *s *of 16% or more (panel b)), while varying the other parameters leads to changes in *s *of no more than about 8%. Changing the minimum age *a*_0 _– below which no malaria infections, and no IPT dosing, was assumed to occur – from 2 months to 1 or 0.5 months made negligible difference to the results. If children aged up to five years are included then the predicted increase in *s *is much greater: 31% (note that is a percent chance in *s*, not the value of *s *itself). The interpretation depends on whether selection is in the earlier, approximately exponential, phase or the later, approximately linear phase of the logistic curve[17]. In the exponential phase, the gene frequency's doubling time is reduced by about 31%. In the linear phase, the time for the frequency to increase any given number of percentage points is reduced by about 31%. The exponential phase occurs at low gene frequency, so in absolute terms, the resistant genotype spreads faster in the linear phase. The Senegalese seasonal IPT scenario results in a predicted increase in *s *of 16%.

## Discussion

The model is intended to yield order of magnitude estimates for different policies, rather than precise values. It makes several simplifying assumptions, but should be much more realistic than using plain drug mass as a measure of selection pressure. It is also limited in that it looks at the immediate impact of IPT on selection pressure, but does not incorporate feedback effects of IPT on parasite prevalence, and hence the tendency of IPT to reduce non-IPT dosings below their baseline value. However, where the impact of selection pressure is small, then these feedback effects would also be small. The model is applicable to combinations such as sulfadoxine-pyrimethamine (Fansidar) or chlorproguanil-dapsone (Lapdap) by using the gene frequency of the set of mutations needed to confer resistance. The half-lives of the single drugs amodiaquine (7–21 days[18]) and mefloquine (14–21 days[19]) are in the range of the sensitivity analysis shown in Figure 2d, which shows their expected results are similar to those of sulfadoxine-pyrimethamine (bearing in mind the assumption that the same drug is used for IPT as for non-IPT therapy). However, when mutations in multiple unlinked genes are needed for resistance, breakdown of their association by recombination will undermine the impact of selection on the spread of resistance. Combination therapy would also be advantageous if the required set of mutations was initially absent in the population, so that selection could not start to act.

Most of these findings are in accordance with those of Prudhomme O'Meara et al[20]: in particular, that the effect of IPTi on selection pressure is likely to be relatively small when treatment is already frequent. Also, their prediction that the spread of fully resistant parasites is likely to be greater in high transmission areas corresponds to that made in the current paper, that the spread will be greater when parasitaemia is more clustered in younger children (since this happens in high transmission areas[21]). Although generally less sophisticated, the current approach does include some features not present in their model: in particular, presumptive treatment of uninfected people outside IPT. We also considered IPT in children older than infants.

The baseline selection coefficient (*s*) estimated for the Tanzanian setting (27%) is towards the high end of the range of 3–33% estimated by Anderson[22] from previously published data on gene frequencies. Tanzania intends to change first line drug treatment to artemether-lumefantrine (CoArtem)[23]. In that situation, the current model would assume that non-IPT treatments would exert no selection pressure on the genotypes associated with resistance to the IPT drug (SP). After introduction, the predicted selection coefficient is 1.1% (approximately equal to the change in selection coefficients when adding IPTi to a background of SP treatment), suggesting that IPTi has little impact on selection pressure in this setting.

Prudhomme O'Meara et al[20] concluded that 'Drugs to which little or no resistance exists are not advisable for IPT in high-transmission areas' because this 'could accelerate the appearance of partial resistance, which could be followed by explosive spread of full resistance'. This assumes that a stepwise accumulation of resistance-associated mutations is the dominant mechanism of the evolution of resistance. However, recent empirical studies have demonstrated that the introduction and spread of alleles of geographically distant origin has been particularly important in the development of resistance to antifolates[24]. This would suggest different advice: that implementation of IPT with a combination of drugs not currently used in the intervention area is most likely to maximize both morbidity reduction and drug lifespan. However, given that 'There is no consensus on how IPT works'[25], further empirical and theoretical work is needed to minimize the impact of resistance on its implementation.

## Conclusion

There are dangers of shortening the useful life of drugs if IPT is implemented over a wide childhood age range. On the other hand, IPT delivered only to infants is unlikely to appreciably shorten the useful life of the drug used.

## Authors' contributions

DS initiated the effort to make quantitative predictions of the effect of IPT on drug resistance, which, with CR, he expressed in terms of the selection coefficient. NA devised the model which was refined with DS, CR and CS, and calibrated by DS and BC. NA wrote the first draft of paper, which was then revised with the others.
